# Best (but oft forgotten) practices: Applying the target trial framework in nutrition research

**DOI:** 10.1016/j.ajcnut.2026.101362

**Published:** 2026-05-22

**Authors:** Michael Fridén, Conor J MacDonald, Deirdre K Tobias, Dalia Stern, Yu-Han Chiu, Daniel B Ibsen

**Affiliations:** 1 Department of Clinical Medicine, Aarhus University, Aarhus, Denmark; 2 Steno Diabetes Center Aarhus, Aarhus University Hospital, Aarhus, Denmark; 3 Novo Nordisk A/S, Bagsværd, Denmark; 4 Institute of Environmental Medicine, Karolinska Institutet, Stockholm, Sweden; 5 Division of Preventive Medicine, Department of Medicine, Brigham and Women’s Hospital and Harvard Medical School, Boston, MA, United States; 6 Nutrition Department, Harvard TH Chan School of Public Health, Boston, MA, United States; 7 SECIHTI-Population Health Research Center, National Institute of Public Health, Cuernavaca, Mexico; 8 Department of Epidemiology, Brown University School of Public Health, Providence, RI, United States; 9 Center for Gerontology and Health Care Research, Brown University School of Public Health, Providence, RI, United States; 10 Department of Public Health, Aarhus University, Aarhus, Denmark

**Keywords:** target trial framework, causal inference, nutrition, epidemiology, randomized trials

## Abstract

When randomized trials in nutrition are not feasible, timely, or ethical, observational studies serve as important pillars of evidence-informed clinical decision-making and public health guidelines. Given that diet is dynamic, multidimensional, and multifactorial, causal questions of dietary exposures are often complex. Consequently, research questions are often poorly defined, making study findings difficult to interpret and implement in the real world. Conceptualizing the design and analysis of observational data like a hypothetical pragmatic randomized trial, a target trial, is one approach to improve the specificity and interpretation of causal questions. Protocol components, including eligibility criteria, treatment strategies, and assignment procedure, outcome and follow-up, causal contrast, and statistical analysis plan, are clearly specified and subsequently implemented using available observational data. In this paper, we explain the target trial framework approach, describe examples from nutrition research, demonstrate the practical steps of performing a target trial study, and discuss the methodological challenges and opportunities of applying the framework to nutrition research.

## Introduction

Estimating causal effects of dietary exposures on health outcomes is a key goal in nutrition research, and randomized controlled trials (RCTs) are regarded as the reference standard study design for obtaining internally valid results. Although well-conducted RCTs should remain the standard to strive for, long-term trials may not always be feasible, timely, or ethical to conduct. Instead, nonrandomized observational studies are often used to complement evidence from RCTs, by, for instance, extending follow-up beyond the typically limited duration of trials, or by including larger populations to increase efficiency for subgroups or secondary outcomes that are underpowered in randomized studies. In addition, even when investments are made to conduct larger-scale dietary RCTs, there is limited follow-up of findings with replication trials. To increase the generalizability of trial findings, therefore, observational studies may be used to emulate existing RCTs in other populations.

Observational data can also be leveraged to address questions that are unlikely ever to be examined by randomizing the intervention, for example, those related to intrauterine nutritional exposures and intake of specific foods for decades across adulthood. However, given that diet is a dynamic, multidimensional, and multifactorial exposure, causal questions of diet addressed in an observational study are inherently complex and often poorly defined [1]. As a result, lack of specificity in the causal question represents one of the major limitations of contemporary observational studies. One approach to improve the design and interpretation of observational studies is to first specify the research question as if it was part of a randomized trial and then emulate that trial using available data. This is the central merit of the target trial framework. When poor alignment between observational and RCT evidence limits the synthesis and translation of their findings for public and patient health decision-making, the target trial framework may help bridge this gap [2]. In this paper, we explain the target trial framework approach, describe examples from nutrition research, demonstrate the practical steps of performing a target trial study, and discuss the methodological challenges and opportunities of applying the framework to nutrition research.

## What Is (Not) the Target Trial Framework?

Designing an observational study to answer a causal question as if it was a randomized trial is not a new concept in epidemiology. However, the concept has been formalized as the “target trial framework” to advance the use of observational data for causal inference and increasingly applied to a wide range of research domains over the last decade [2]. The main objective of the framework is to approach the design and analysis of observational data as you would for a hypothetical pragmatic RCT; the RCT that you would have liked to conduct had it been feasible to do so (the “target trial”). This includes establishing key components of an RCT protocol, such as: Who would be eligible to participate? What would the precise treatment strategies be, and how would you assign participants to these strategies? When would participants initiate the treatment strategies, and would they be fixed for everyone, or would some be allowed to discontinue or modify the strategies on developing an intolerance or other contraindications over follow-up? What is the causal contrast you are interested in, and how would you analyze the data to estimate it? Such essential parts of the study design are established before enrolling the first participant in a randomized trial, but seemingly less consideration is given to these when asking causal questions from observational data. When the hypothetical target trial has been specified, researchers subsequently aim to emulate the trial by mapping each protocol component to the observed data. In light of this, the target trial framework is not a “statistical method,” as sometimes alluded to, but a framework for an analysis plan that can be applied across all fields of epidemiology that aim to estimate causal effects from observational data.

**FIGURE 1:**
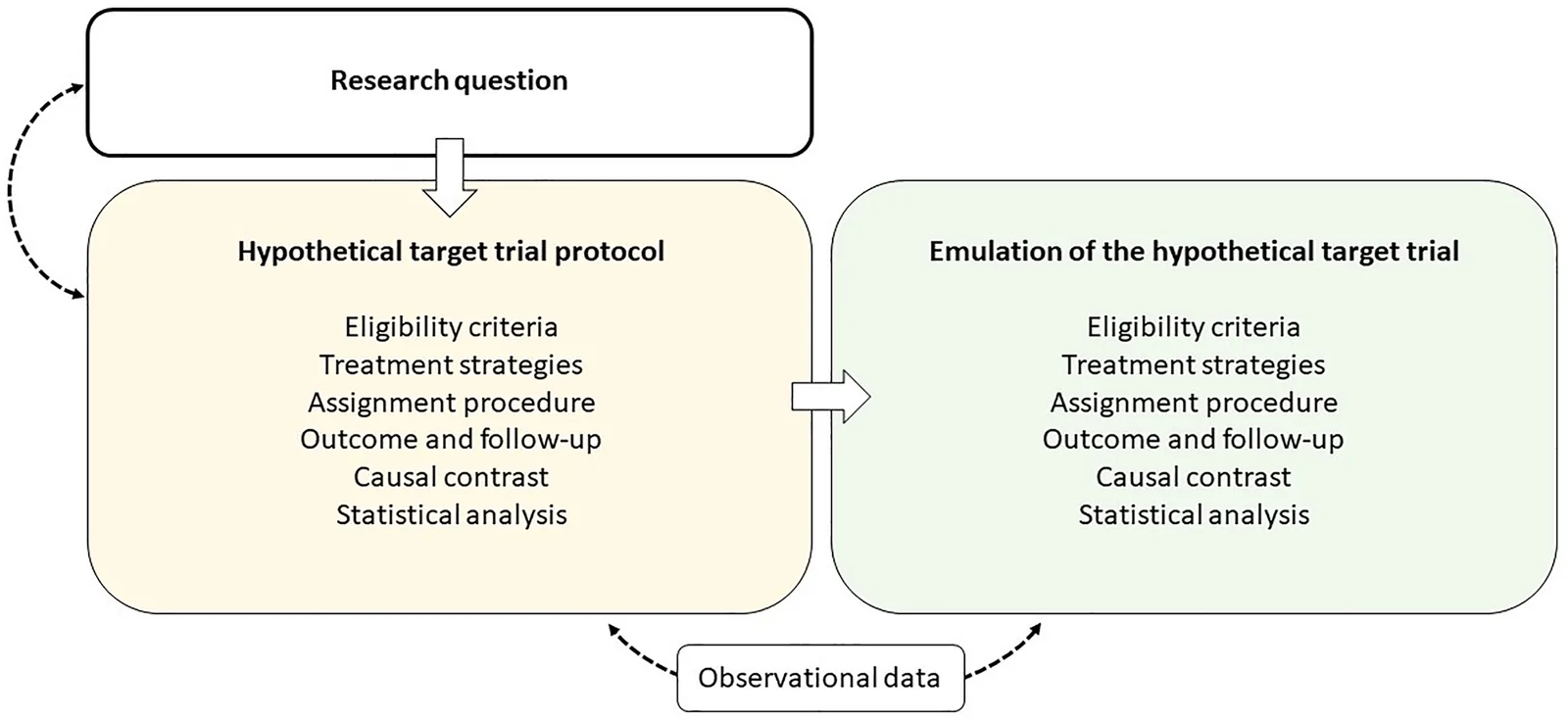
An illustration of the iterative process underlying emulating a hypothetical pragmatic target trial using observational data. The research question you initially want to answer naturally leads to the specification of a hypothetical target trial protocol, by which you then try to emulate using observational data. The dotted lines with bidirectional arrows indicate that formulating a well-defined causal question that can be answered using observational data is an iterative process. If the available data do not allow you to answer your original question, the question may be adapted.

Ultimately, the target trial framework helps the investigator make the causal question explicit, thereby setting up the analysis accordingly, and contributing findings that are interpretable and applicable to the broader evidence base and subsequent real-world decision-making. In practice, emulating a hypothetical target trial is an iterative process in which the original research question may need to be transparently modified to align with the available data (Figure 1 describes the underlying process). Importantly, a target trial is not an ideal trial, but a trial that can be reasonably emulated with existing data [3,4]. Similarly, a target trial is not an explanatory trial, but a pragmatic trial where participants, treatment prescribers, and investigators are aware of participants’ allocated treatment.

### Examples from cohort studies implementing the target trial framework in nutrition

So far, there have been a limited number of published studies of nutritional exposures explicitly stating they used the target trial framework [[5], [6], [7], [8], [9], [10], [11], [12]] (or similar hypothetical intervention approaches [[13], [14], [15]]) for their observational analyses, summarized in Table 1 [[5], [6], [7], [8], [9], [10], [11], [12], [13], [14], [15]]. Most studies compared a dietary pattern intervention, food or joint diet strategy to “no intervention.” The “no-intervention” arm is equivalent to a control group being assigned to continue their usual diet, which has also been a common control group in previously conducted RCTs. In contrast, Lajous et al. [14], Chiu et al. [5], Gebremariam et al. [15], and Elahy et al. [9] used active comparator designs. Except for Elahy et al. [9] and Gebremariam et al. [15], the analyses were conducted in observational studies that had repeated measures of diet. The importance and potential challenges with analyses of repeated exposure measures will be discussed later.

**TABLE 1:** A summary of some target trial emulation studies in nutrition

Study	Causal question^1^	Observational data used to emulate the target trial	Findings^1^
Danaei et al., 2013 [13]	What are the causal effects of sustained adherence to various hypothetical lifestyle interventions (including reducing red and processed RM and soda, and increasing coffee, alcohol, and WG) as compared with no intervention, on the 24-y risk of type 2 diabetes in females during midlife?	Country: United States Cohort: NHS	RD (95% CI): RM: −0.8% (−1.0%, −0.5%) Soda: −0.2% (−0.6%, 0.2%) Coffee: −0.3% (−0.6%, 0.0%) Alcohol: −1.8% (−2.3%, −1.2%) WG: −0.05% (−0.4%, 0.3%) Jointly: −2.8% (−3.4%, −2.0%) RR (95% CI): RM: 0.92 (0.89, 0.95) Soda: 0.98 (0.94, 1.02) Coffee: 0.97 (0.94, 1.00) Alcohol: 0.81 (0.77, 0.88) WG: 0.99 (0.96, 1.04) Jointly: 0.71 (0.65, 0.79)
Lajous et al., 2013 [14]	What are the causal effects of sustained adherence to *1)* ≥1 serving of fish/wk, *2)* ≥2 servings of fish/wk, *3)* ≥3 servings of fish/wk, or *4)* ≥5 servings of fish/wk, as compared with 0 servings of fish/wk, on the 18–22-y risk of total coronary artery disease in males and females?	Country: United States Cohorts: HPFS and the NHS	RR (95% CI): 1 serving of fish/wk HPFS: 1.02 (0.92, 1.12) NHS: 0.91 (0.83, 1.00) 2 servings of fish/wk HPFS: 1.03 (0.90, 1.15) NHS: 0.87 (0.76, 0.98) 3 servings of fish/wk HPFS: 1.06 (0.92, 1.21) NHS: 0.82 (0.67, 0.95) 5 servings of fish/wk HPFS: 1.08 (0.86, 1.29) NHS: 0.82 (0.61, 1.07)
Chiu et al., 2020 [5]	What are the causal effects of maintaining sugar-sweetened beverage consumption at or <0.5 servings/d, as compared with >0.5 servings/d, during pregnancy and early childhood (PC), during pregnancy only (P), or during early childhood only (C), on early adolescent BMI *z*-score?	Country: United States Cohort: Project Viva	MD (95% CI): PC: −0.94 units (−1.52, −0.08) P: −0.05 units (−0.34, 0.23) C: −0.89 units (−1.46, −0.11)
Gebremariam et al., 2021 [15]	What are the causal effects of jointly or separately increasing FV to >5 servings/d compared with <5, BF consumption to once/d compared with none, and reducing SSB consumption to 0 times/d compared with >0 times/d, in 11-y-old adolescents on BMI at age 13?	Country: Norway Cohort: HEIA	MD (95% CI): Jointly: −0.21 kg/m^2^ (−0.48 kg/m^2^, 0.10 kg/m^2^) FV: −0.08 kg/m^2^ (−0.30 kg/m^2^, 0.13 kg/m^2^) BF: −0.01 kg/m^2^ (−0.13 kg/m^2^, 0.13 kg/m^2^) SSB: −0.12 kg/m^2^ (−0.36 kg/m^2^, 0.14 kg/m^2^)
Chiu et al., 2021 [6]	What is the causal effect of sustained adherence to the AHA 2020 dietary goals, as compared with no intervention, on the 20-y mortality risk in adult males and females?	Country: United States Cohorts: The HPFS, the NHS I and II	RD (95% CI): HPFS: −3.9% (−4.9%, −3.2%) NHS I: −2.6% (−3.1%, −1.8%) NHS II: −0.35% (−0.56%, −0.09%) RR (95% CI): HPFS: 0.85 (0.81, 0.88) NHS I: 0.79 (0.75, 0.85) NHS II: 0.86 (0.78, 0.96)
Ibsen et al., 2024 [7]	What is the causal effect of long-term adherence to the DASH diet, as compared with no intervention, on the 22-y risk of heart failure in middle-aged Swedish males and females?	Country: Sweden Cohorts: The SMC and the COSM	RD (95% CI): SMC: −0.7% (−1.6%, 0.0%) COSM: −0.9% (−1.6%, −0.2%) RR (95% CI): SMC: 0.95 (0.90, 1.00) COSM: 0.95 (0.90, 0.99)
Guo et al., 2025 [8]	What are the causal effects of sustained adherence to the World Cancer Research Fund/American Institute of Cancer Research’s PA and dietary guidelines (independently and jointly), as compared with no intervention, on the 26-y risk of total prostate cancer in males?	Country: United States Cohort: The HPFS	RD (95% CI): PA: 0.03% (0.02%, 0.05%) Diet: 1.84% (−0.64%, 4.21%) Jointly: 1.87% (−0.61%, 4.25%) RR (95% CI): PA: 1.00 (1.00, 1.00) Diet: 1.11 (0.96, 1.26) Jointly: 1.11 (0.96, 1.26)
Elahy et al., 2025 [9]	What is the causal effect of *1)* sustaining ≥12 h of overnight fasting, as compared with <12 h, *2)* sustaining ≥4 h of fasting before sleep, as compared with <4 h, *3)* sustaining ≥1 h or fasting after sleep, as compared with <1 h, for 12 mo on mean body weight 2 y post baseline in United States adults across 3 independent interventions?	Country: United States Cohort: CPS-3 DAS	MD (95% CI): 12 h: 0.4 kg (−4.1 kg, 4.7 kg) 4 h: 1.9 kg (−0.4 kg, 4.1 kg) 1 h: 0.9 kg (−4.3 kg, 4.4 kg)
McGee et al., 2025 [10]	What is the causal effect of sustained adherence to 7 physical activity and dietary recommendations adapted from the 2018 World Cancer Research Fund/American Institute for Cancer Research and 2022 ACS lifestyle recommendations for cancer survivors, as compared with no intervention, on the 20-y mortality risk in adult breast- and prostate cancer survivors?	Country: United States Cohorts: The HPFS (males), the NHS I and II (females)	RD (95% CI): NHS: −9.5% (−11.7%, −7.2%) HPFS: −9.2% (−12.5%, −5.4%) RR (95% CI): NHS: 0.70 (0.63, 0.78) HPFS: 0.81 (0.73, 0.89)
Harnois-Leblanc et al., 2025 [11]	What are the sex-specific effects of limiting sugar-sweetened beverages to no >1 serving/wk or limiting 100% fruit juice to no >1 serving daily throughout childhood, as compared with no intervention, on HOMA-IR?	Country: United States Cohort: Project Viva	MD (95% CI): Females: 0.07 units (−0.13, 0.29) Males: −0.28 units (−0.61, 0.02)
Stern et al., 2026 [12]	What is the effect of adopting the 2023 Mexican Dietary Guidelines, as compared with no intervention, on the 9-y risk of type 2 diabetes in females?	Country: Mexico Cohort: The Mexican Teachers Cohort	RD (95% CI): −1.33% (−3.21%, 1.29%) RR (95% CI): 0.84 (0.61, 1.15)

Results are presented as MD for continuous outcomes and as RD in percentage points (%) and RR for survival outcomes.

Abbreviations: AHA, American Heart Association; BF, breakfast; CI, confidence interval; COSM, Cohort of Swedish Men; DASH, Dietary Approaches to Stop Hypertension; FV, fruits and vegetables; HEIA, HEalth In Adolescents; HPFS, Health Professionals Follow-up Study; MD, mean differences; NHS, Nurses’ Health Study; PA, physical activity; RD, risk differences; RM, red meat; RR, risk ratios; SMC, Swedish Mammography Cohort; SSB, sugar-sweetened beverage; WG, whole grains; CPS-3 DAS, Cancer Prevention Study-3 Diet Assessment Substudy; ACS, American Cancer Society.

1 The causal questions shown in Table 1 do not necessarily correspond to the exact research questions as formulated in the original studies; rather, they represent our interpretation of the underlying causal aims based on the presented target trial protocols (or similar hypothetical intervention strategies). Due to limited space, we chose to only show results for some, but not all, of the research questions from the included studies (e.g., Lajous et al. [14] also estimated the effects of substituting red meat with fish, and McGee et al. [10] also estimated the effects of reducing alcohol consumption).

Few studies compared their target trial emulation results to results from conventional approaches. Ibsen et al. [16] estimated the effect of adhering to an established Dietary Approaches to Stop Hypertension (DASH) diet pattern on the risk of heart failure using traditional observational epidemiology approaches, and later conducted an updated analysis using a target trial emulation in the same Swedish cohorts. The hazard ratio from the conventional analysis was modestly stronger (hazard ratio: 0.83) than the risk ratio estimated from the target trial emulation (risk ratio: 0.95) [7]. One explanation for the incompatible results was that the 2 studies compared different dietary patterns with different comparison diets. The conventional study compared participants with the highest DASH score, although no one perfectly followed all the components simultaneously, to those with the lowest score. The target trial study, on the other hand, estimated the risk under a different scenario; specifically, when everyone in the population followed all components jointly of a population-adapted DASH diet compared with continuing with their usual diet. In addition, the lack of appropriate statistical methods used to account for time-varying dietary intake and confounders (discussed later) in the conventional analysis may also have contributed to the incompatible results.

## Methodological Challenges and Potential Solutions for Estimating Causal Effects of Dietary Exposures from Observational Data

In this section, we outline key methodological challenges in estimating causal effects from observational nutritional data and discuss potential solutions. These challenges are not specific to the use of the target trial framework, but nevertheless important to consider when specifying and emulating a target trial. A summary of the section is presented in Table 2 [[17], [18], [19], [20], [21], [22]].

**TABLE 2:** A summary of methodological challenges, potential solutions, and opportunities of implementing the target trial framework in nutrition research

Methodological challenges	Potential solutions
Confounding, selection bias, measurement error, and model misspecification	Measurement error: Use reliable measurement tools, continue to put effort into improving the precision and accuracy of dietary assessment methods (e.g., by identifying dietary biomarkers), and/or use bias adjustment techniques such as regression calibration. Confounding, selection bias, and model misspecification: Conduct sensitivity analyses by using negative control outcomes [17], adjusting for previous dietary variables (and other covariates) [7,10], changing the functional form of confounders, and comparing the natural risk under no intervention with the risk obtained from the parametric estimators [18,19] or use nonparametric or more flexible estimators (e.g., targeted maximum likelihood estimation with machine learning algorithms) [20].
Multiple versions of treatment	Define the interventions (including the comparator) sufficiently well so that no relevant multiple versions of treatment exist. Describe the assumptions underlying the definitions of the intervention strategies.
Potential positivity violations	Make sure there are individuals adhering to the interventions at each time point. The positivity assumption is the only identifiability assumption that can, at least to some extent, be evaluated empirically. If not, modify the intervention strategies to avoid potential positivity violations [7,12].
Previous diet as a potential confounder	If possible, change baseline to a later time point to adjust for prebaseline diet (e.g., in the simulated example, start baseline in 2010 and adjust for dietary intake in 2005). If data are unavailable, conduct sensitivity analyses to examine robustness to confounding or quantitative bias analyses to quantify the potential confounding bias [7,21].
Lack of repeated measures of diet and confounders	If possible, collect rich and repeated measures of diet and confounders when designing the cohort study. When repeated measures are lacking, explain why sustained adherence may reasonably be approximated using only 1 dietary assessment, for example, in studies with short follow-up.
The use of more sophisticated statistical tools is required when estimating sustained adherence to diets in the presence of treatment-confounder feedback	Continue to publicly share code, develop user-friendly packages and macros in commonly used statistical software, invest more time and effort in educating causal inference methods in nutrition, and emphasize transparency of the methods and underlying assumptions in future studies (the new TrAnsparent ReportinG of observational studies Emulating a Target trial (TARGET) guidelines may be used for guidance [22]).
Prevalent users and the background diet	Describe and acknowledge the background diet for correct interpretation and comparison across studies.

Opportunities

By formulating a well-defined causal question anchored in a hypothetical target trial, the resulting estimates become easier to interpret and more directly applicable for informing public and patient health decisions.

### Confounding, selection bias, measurement error, and model misspecification

In general, estimating causal effects of diet on health outcomes from observational studies relies on strong assumptions, including no unmeasured confounding, no selection bias, no measurement error, and no model misspecification. None of these assumptions will be inherently satisfied by using the target trial framework. As such, continued efforts to improve the precision and accuracy of dietary assessment tools to estimate long-term habitual diet, as well as implementing carefully conducted sensitivity analyses or quantitative bias analyses to examine the robustness of the results, will continue to be important. Although misspecification of time zero (i.e., the misalignment of eligibility criteria, treatment assignment, and start of follow-up) is typically known to introduce time-related biases in fields like pharmacoepidemiology [23], similar concerns arise in nutritional epidemiology. This issue is especially salient in studies that evaluate changes in intake and relate them to subsequent outcomes, where failure to clearly define and align time zero may introduce bias. By aligning eligibility criteria, treatment assignment, and start of follow-up, as would have been done in a randomized trial, issues such as these would be mitigated.

### Defining the dietary interventions

In contrast to placebo-controlled pharmaceutical RCTs with standardized formulations and dosing regimens, dietary interventions are dynamic, multidimensional, and multifactorial, and in most cases lack a placebo [1]. This complexity poses unique challenges for clearly specifying the intervention and comparator of interest. Furthermore, in conventional observational analyses, the common practice of aggregating many different foods into dietary pattern scores or quantiles may give rise to multiple relevant versions of treatment, posing additional challenges in interpretation [24]. For example, imagine an observational study aiming to estimate the effect of a low-carbohydrate diet score on the risk of type 2 diabetes. Researchers start by grouping participants into quartiles based on the proportion of carbohydrates constituting the individual’s diet and move on to estimate relative risks comparing the 2 end-quartiles. If cohort participants are allowed to arrive at the highest quartile through a range of different low-carbohydrate foods, results from this analysis would probably be difficult to interpret and hard to use to inform decisions of what to eat in the real world. Similarly, if the distributions of the food groups differ between populations, effect estimates will also likely vary. Therefore, the intervention strategy would benefit from further specification, by, for example, defining intake thresholds for specific low-carbohydrate foods (e.g., processed red meat and green-leafy vegetables), thereby mimicking what would have been done in a well-planned randomized trial. Importantly, because participants may end up in the lowest quartile in many different ways, the comparator would also need to be more clearly defined. For public health relevance, the comparator could be specified as the usual diet (i.e., no intervention) to estimate the effect had the population shifted their diet to eat fewer carbohydrate-rich foods. For comparative effectiveness, an active dietary intervention would be a relevant comparator (e.g., the Mediterranean diet). Although a key strength of the target trial framework lies in reducing vagueness in the definition of interventions, some ambiguity remains about the level of precision required. Whether “processed red meat” or “green-leafy vegetables” as composite food groups are sufficiently well defined to produce interpretable estimates is highly dependent on the context of the study and current scientific consensus [25].

Another potential challenge is that of positivity. Positivity refers to the nonzero probability of receiving either intervention. In a randomized trial, the probability of assignment (but not adherence) to a dietary intervention is determined by the investigator during the design phase via the randomization scheme (e.g., 1:1 allocation ratio). In an observational study, however, the presence of the dietary exposure may need to be verified first. If the food of interest was rarely consumed by participants in the study population, then it will not be possible to estimate its effect on a health outcome. The risk of violating the positivity assumption may be greater when defined strategies are complex, involving many different foods and time points. Researchers may then have to modify the interventions and conduct sensitivity analyses to examine robustness to potential positivity violations [7,10,12]. For example, in [12], the investigators required that ≥10% of the study population would meet the recommendations for each of the food groups defined in the 2023 Mexican Dietary Guidelines. As such, they had to adapt the thresholds for several of them and transparently acknowledge that the original research question changed as a consequence.

Lastly, for research questions involving food substitutions, such as replacing 1 serving of red meat with 1 serving of fish, one must be explicit about what is being substituted, at what levels, over what time frame, and how the substitution is sustained during that period, particularly in longitudinal settings where diet changes over time. Without careful specification, it becomes unclear what causal contrast is being estimated. Interested readers are referred to [20,26] for more comprehensive discussions on emulations involving food substitutions.

### Lack of data on prebaseline diet

In an RCT, randomization ensures that prebaseline foods are equally distributed across diet groups. When estimating effects from observational data, however, be it from some hypothetical dietary intervention or some score-based exposure, failing to account for prebaseline diet would likely introduce confounding bias, as prior diet may influence both current dietary habits as well as the outcome of interest. Consequently, obtaining valid effect estimates from observational data typically requires repeated and frequently updated dietary measurements (≥2) for all eligible participants, a resource that is scarce in most existing cohorts. When unavailable, researchers are encouraged to perform sensitivity analyses, for example, by including previous diet as a confounder in a subpopulation, as was done by Ibsen et al. [7] or conducting tipping point analyses [21].

### Diet as a time-varying exposure

**FIGURE 2:**
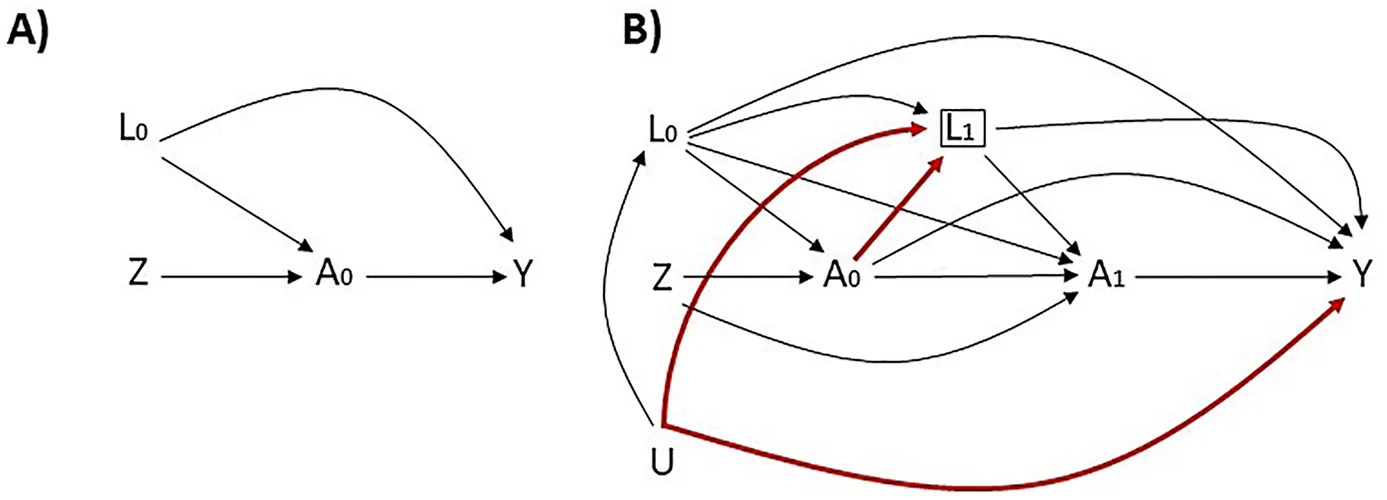
Two simplified directed acyclic graphs [29] depicting the causal assumptions underlying valid estimation of (A) the intention-to-treat effect and (B) the per-protocol effect of sustained adherence. Readers not familiar with directed acyclic graphs are referred to Tennant et al. [29] for an in-depth review. In short, these graphs are graphical tools with a set of mathematical rules that help researchers draw their causal assumptions and to identify biasing pathways. (A) The intention-to-treat effect is the effect of assignment (in this study, through randomization). Let Z be the assignment to the diets, A0 be adherence to the diets at baseline, L0 be a vector of baseline confounders, and Y be the outcome. We assume no loss to follow-up. For simplicity, and perhaps unrealistically in a pragmatic trial, we assume Z to only affect the outcome through A. Estimating the effect of Z on Y (i.e., the intention-to-treat effect) requires no adjustment for confounding as diets are randomly assigned. (B) The per-protocol effect is the joint effect of adhering to the assigned diets. Let A1 be adherence to the diets at time point 1, L1 be a vector of confounders at time point 1, and U represent some unmeasured confounders. We assume no loss to follow-up and that Z only affects the outcome through A. To estimate the effect of Z, A0, and A1 on Y (i.e., the per-protocol effect), one must address confounding at both baseline and at time point 1. However, using conventional statistical approaches to adjust for L1 (indicated by the square around L1) would 1) block the indirect pathway from A0 to L1 to Y but also 2) in accordance with causal graph theory “open up a backdoor path,” meaning creating a biasing path, between A0 and L1 to U and Y (indicated by the red pathway). In the presence of treatment-confounder feedback (A0 to L1 to A1 in our example), we need to use g-methods to appropriately account for this.

A common causal contrast of interest from previous studies (Table 1) was the effect of sustained adherence to the assigned dietary strategy over follow-up, commonly referred to as the observational analog of the per-protocol effect [27] (strictly speaking, interpreting this contrast as the per-protocol effect requires additional assumptions; see [28]). This causal contrast is different from the one most often targeted in RCTs: the intention-to-treat effect. Although the per-protocol approach estimates the effect of adhering to the assigned diet protocol—a joint intervention not to be conflated with the effect of treatment [28]—the intention-to-treat approach estimates the effect of assignment, regardless of adherence [27]. Although the former contrast may be more relevant for real-world decision making in the presence of low adherence, per-protocol effect estimation relies on strong assumptions of no unmeasured confounding at each time point during follow-up [27]. In longitudinal settings with time-varying diet and treatment-confounder feedback (e.g., when prior diet affects BMI, which in turn influences subsequent dietary adherence; see Figure 2 [29] for a graphical illustration of the treatment-confounder feedback issue) [30], appropriate estimation typically requires g-methods, such as inverse probability weighting, parametric g-computation, or doubly robust approaches like targeted maximum likelihood estimation. Limited familiarity with these methods may partly explain their relatively infrequent use in nutritional epidemiology, although increasing availability of user-friendly software in R and SAS is improving accessibility [27, CAUSALab | Harvard T.H. Chan School of Public Health].

However, validly estimating effects of time-varying, and sometimes dynamic (e.g., when a decision to intervene is dependent on some evolving characteristic), interventions in both randomized trials and observational studies generally require rich repeated data on both diet and confounders, a feature that is lacking for most existing cohorts. When diet is assessed only once, researchers have to make additional assumptions when claiming to estimate causal effects of some hypothetical sustained interventions on some health outcomes. First, adherence to the protocol is assumed to be sustained from the point of assignment until the end of intervention. Second, the researcher would have to assume that the prebaseline diet is not a confounder for the specified exposure–outcome relation. The first assumption would be more likely to hold should the intervention period be relatively short. The assumption of no confounding by prebaseline diet is nevertheless stronger, even for short-term studies, and sensitivity analyses are encouraged to quantify the potential confounding bias [7,21].

### Prevalent users and the background diet

Lastly, an often-overlooked aspect of nutritional research that distinguishes the nutrition field from, for example, clinical pharmacology, is the fact that individuals are constantly exposed to food. Researchers are seldom able to intervene on treatment-naïve participants, as would be feasible in clinical trials of pharmacological treatments. The diet consumed on entering a trial, referred to as the background diet, is therefore highly important to acknowledge and, if possible, to thoroughly describe. To see why, consider an investigation aimed at estimating the effect of consuming ≥3 servings of fish/wk, compared with no intervention, on LDL cholesterol concentrations. If the baseline distribution of fish intake differs between 2, otherwise, virtually identical populations, the corresponding effect estimates will differ as well. This is simply because fish intake entering a trial determines the absolute change required to meet the dietary goal of 3 servings of fish/wk in the treatment arm, with greater changes leading to larger effects. In addition, the magnitude and direction of the effect on LDL cholesterol will depend on the distribution of foods in the no-intervention arm. The background diet therefore has immediate implications for the interpretation of the results and the extent to which the results can be transported across populations [1].

## How to Get Started? An Example of the Nordic Nutrition Recommendations 2023 and All-Cause Mortality

In this study, we provide a simple synthetic example of how to specify and emulate a hypothetical target trial. The process uses a 2-step approach: defining the protocol of the hypothetical target trial (step 1) and emulating the target trial protocol using available data (step 2). Each step is further divided into 6 intermediary steps, representing the different protocol components ranging from eligibility criteria to the statistical analysis plan (Table 3). We provide a condensed practical guide in the main manuscript. The reader is referred to Supplementary Material for detailed information on the 6 intermediary steps and the accompanying R-code.

**TABLE 3:** Specification and emulation of the target trial protocol followed by general points of consideration

Protocol components	Hypothetical target trial	Emulation using simulated observational data	General points of consideration
Eligibility criteria	Males and females ≥45 but ≤60 y of age.	Same. All individuals are between 45and 60 y of age. Participants were also required to have complete and valid information on FFQ data and other covariates at baseline.	Are all individuals between the ages of 45 and 60 eligible to participate? What about those who cannot adhere to the interventions, e.g., individuals with gastrointestinal issues, vegans, or a nut allergy?
Treatment strategies	Participants would be randomly assigned to either the NNR23 or no intervention. The food thresholds in NNR23 would be: F&V: ≥500 g/d RPM: ≤350 g/wk Nuts: ≥20 g/d At each 6-mo follow-up meeting, individuals would be encouraged to continue to eat according to the intervention until the next scheduled visit. The no-intervention group would continue as usual.	Same as the target trial + the assumption that reported intake from FFQs represents the mean intake until answering the next FFQ, loss to follow-up, or end of follow-up. The no-intervention arm will assume that individuals eat according to their reported intake from the FFQ. If the natural value of intake falls below or above the recommended value, it would be changed to a value compatible with the intervention under each specified strategy. Instead of biannual follow-up meetings, participants are followed up every 5 y.	Is the assumption of sustained adherence between each follow-up reasonable? Are all participants intervened on? What about those who develop gastrointestinal issues or an allergy to nuts? Are there people in the data actually adhering to these intervention thresholds? If not, thresholds probably need to be adapted to the population. What is the composition of the diet in the no-intervention group? This would be important to describe for correct interpretation and comparison across studies.
Assignment procedure	Each participant would be randomly assigned (in a 1:1 ratio) to either the NNR23 or to no intervention in 2005. Participants would be aware of their allocated diet.	We assume randomization based on BMI, sex, and education at baseline in 2005.	Are there any other baseline confounders that need to be adjusted for? What about prebaseline dietary variables and/or prebaseline covariates? If all confounders are measured at the same time as the exposure, this needs to be considered.
Outcome and follow-up	The outcome would be all-cause mortality. Participants would be followed up from baseline in 2005 to event, loss to follow-up, or administrative end of follow-up in 2025.	Same. Loss to follow-up was defined as not returning valid FFQ data.	Are there any competing events that need to be considered? How are they handled? For all-cause mortality, this is not an issue.
Causal contrast	Per-protocol effect.	Observational analog of the per-protocol effect.	Is there treatment-confounder feedback present for the estimation of the per-protocol effect? If so, g-methods are required.
Statistical analysis	Apply the parametric g-formula, with adjustment for prebaseline and postbaseline variables associated with adherence, loss to follow-up, and the outcome.	Same. Baseline sex and education, as well as BMI as a time-varying covariate, will be adjusted for.	Are there other important time-varying covariates predictive of adherence and/or loss to follow-up? Are the assumptions of no unmeasured confounding, no measurement error, no selection bias, and no model misspecification reasonable? Consider sensitivity analyses to examine robustness to these assumptions.

Abbreviations: FFQ, Food Frequency Questionnaire; F&V, fruits and vegetables; NNR23, Nordic Nutrition Recommendations 2023; RPM, red and processed red meat.

Let us suppose that we are interested in the following causal question: *what is the effect of sustained adherence to the Nordic Nutrition Recommendations 2023 (NNR23), as compared with no intervention, on the 20-y risk of all-cause mortality in Swedish middle-aged males and females?* By using the target trial framework to answer such a question, we first need to define the protocol for the hypothetical target trial that we would have liked to conduct had it been feasible to do so. This is step 1. Next, we use our observational data to emulate each component of the protocol. This is step 2. The intermediary steps 1.1 to 1.6 and 2.1 to 2.6 are shown in Table 3 and in Supplementary Material. For the purpose of this exercise, we simulated a population of *n* = 10,000 males and females aged 45 to 60 y of age at the time of recruitment in 2005. Repeated measures of diet, captured through validated food frequency questionnaires, and confounders were assessed every 5 y (2005, 2010, 2015, 2020, and 2025). For simplicity, we simulated complete data on all covariates and no loss to follow-up. We also defined the NNR23 diet based on 3 food groups only: fruits and vegetables, red and processed red meat, and nuts. To estimate sustained adherence to the NNR23, i.e., the per-protocol effect, in the presence of treatment-confounder feedback (e.g., BMI and diet), the parametric g-formula was used for the statistical analysis. For a detailed description of the parametric g-formula, the reader is referred to the paper by McGrath et al. [18]. Other g-methods, such as targeted maximum likelihood estimation, would also suffice. Once the data have been structured in long format (with each time interval for each ID separated by rows), eligibility criteria, number of time points, intervention names and thresholds, baseline and time-varying confounders and their functional forms, as well as outcome and covariate models are specified before running the g-formula.

From the example, we estimated an absolute 20-y mortality risk of 47.3% [95% confidence interval (CI): 46.3%, 48.3%] had everybody in the population continued as usual (no intervention), and 44.0% (95% CI: 42.0%, 45.8%) had everybody in the population adhered to the assigned NNR23 over follow-up. The mean proportion of individuals who would have been required to change their diet at each time point to adhere to the assigned NNR23 strategy was 77.4%. Compared with no intervention, we estimated an absolute risk reduction of −3.3 percentage points (95% CI: −5.2, −1.9) and a risk ratio of 0.93 (95% CI: 0.89, 0.96). Assuming no unmeasured confounding, no measurement error, no selection bias, and no model misspecification, these estimates correspond to the causal effect of sustained adherence to the NNR23, as compared with no intervention, on the 20-y risk of all-cause mortality in Swedish middle-aged males and females. However, these assumptions are strong and need to be thoroughly examined. As an example, we purposely made the strong, and possibly unreasonable, assumption that the previous diet is not a relevant confounder. A more valid approach would have been to change the baseline to 2010 to allow for adjustment for the prebaseline diet in 2005. To evaluate the potential for model misspecification, we compared the nonparametric risk estimates under no intervention with the parametric g-form risk estimate. The 2 were very similar, which supports, but does not guarantee, the absence of gross model misspecification (Supplementary Material) [[18], [19]]. Other commonly implemented sensitivity analyses for the studies in Table 1 include the use of negative control outcomes [17], adjustment for previous dietary variables in a subset of the population [7], changing the functional forms of the covariates, as well as changing the order of the covariates when specifying the covariate models in the parametric g-formula. There are, however, other sensitivity analyses and quantitative bias analyses that could be used to examine robustness to these assumptions [10,21].

## The Target Trial Framework Offers Opportunities to Improve Nutrition Science

The target trial framework is not intended to replace or compete with well-conducted randomized dietary trials. Instead, observational studies may effectively complement evidence from trials by, for example, extending follow-up beyond the typically limited duration of trials, or by including larger populations to improve generalizability or increase efficiency for subgroups or secondary outcomes that are underpowered in randomized studies. Likewise, randomized trials can effectively complement observational studies by serving as a benchmark against which the validity of the target trial emulation can be assessed [31]. If results from an emulated trial align with existing results from a real randomized trial (e.g., adherence to the Mediterranean diet on the 2-y mortality risk), one can be more confident that an estimate from an extension of the same emulated trial (e.g., adherence to the Mediterranean diet on the 5-y mortality risk) represents a causal effect. Furthermore, bridging the observational and the randomized interventional design by conceptualizing a hypothetical trial that one wishes to conduct forces researchers to clearly define the causal question under investigation, when randomized trials are not feasible. If causal questions are not well defined, which they may not always be in nutrition [1], the numerical quantity generated from the statistical analysis will not be either. This is a fundamental concept in causal inference, and the target trial framework helps researchers to avoid, or at least reduce, this vagueness. By using the framework, causal questions are made explicit to all stakeholders involved, and as a result, estimates become easier to interpret and thus more informative for public and patient health decision making [1].

## Author contributions

The authors’ responsibilities were as follows – MF, DBI: initiated the idea of the project; MF, CJM, DKT, DS, Y-HC, DBI: designed the research; MF: wrote the first draft of the manuscript; MF: had primary responsible for final content; and all authors read and approved the final manuscript.

## Data availability

R-code and the generated data for the synthetic example presented in this perspective article are available in Supplementary Material.

## Declaration of Generative AI and AI-Assisted Technologies in the Writing Process

During the preparation of this work, the authors used ChatGPT to improve language and modify parts of the code. After using this tool/service, the authors reviewed and edited the content as needed and take full responsibility for the content of the published article.

## Funding

MF is supported by a research grant from the Danish Diabetes and Endocrine Academy, which is funded by the Novo Nordisk Foundation, grant number NNF22SA0079901. DBI is funded by the Novo Nordisk Foundation, grant number NNF24SA0097844, and the Steno Diabetes Center Aarhus, which is partially funded by an unrestricted donation from the Novo Nordisk Foundation. Y-HC is supported by the National Institute of General Medical Sciences of the National Institutes of Health under award number R35GM154888. The sponsors had no role in the study design, data collection, interpretation of data, writing of the report, review, approval, or submission of the article for publication.

## Conflict of interest

MF reports financial support was provided by Danish Diabetes and Endocrine Academy. DBI reports financial support was provided by Novo Nordisk Foundation and Steno Diabetes Center Aarhus. Y-HC reports financial support was provided by National Institute of General Medical Sciences of the National Institutes of Health. CJM reports a relationship with Novo Nordisk that includes: employment. DKT is an Editor for the *American Journal of Clinical Nutrition* and played no role in the Journal’s evaluation of the manuscript. The other authors report no conflicts of interest.
