# Supplementary material for: Best (but oft forgotten) practices: Applying the target trial framework in nutrition research
Source: Am J Clin Nutr. 2026 May 22;124(1):101362. doi: 10.1016/j.ajcnut.2026.101362 (PMC13494081; doi:10.1016/j.ajcnut.2026.101362)
Supplement: Supplementary file 1 [file mmc1.docx]

**Best (but oft forgotten) practices: Applying the target trial framework in nutrition research

Supplementary Material**

Michael Fridén^1,2^, Conor J. MacDonald^3,4^, Deirdre K. Tobias^5,6^, Dalia Stern^7^, Yu-Han Chiu^8,9^, Daniel B. Ibsen^2,10^

¹Department of Clinical Medicine, Aarhus University, Aarhus, Denmark.
²Steno Diabetes Center Aarhus, Aarhus University Hospital, Aarhus, Denmark.
^3^Novo Nordisk A/S.
^4^Institute of Environmental Medicine, Karolinska Institutet, Stockholm, Sweden.
^5^Division of Preventive Medicine, Department of Medicine, Brigham and Women's Hospital and Harvard Medical School, Boston, MA, USA.
^6^Nutrition Department, Harvard TH Chan School of Public Health, Boston, MA, USA.
^7^SECIHTI-Population Health Research Center, National Institute of Public Health, 62100 Cuernavaca, Mexico.
^8^Department of Epidemiology, Brown University School of Public Health, Providence, RI, 02903, USA.
^9^Center for Gerontology and Health Care Research, Brown University School of Public Health, Providence, RI, 02903, USA
^10^Department of Public Health, Aarhus University, Aarhus, Denmark.

A step-by-step tutorial with accompanied R-code for a target trial emulation of sustained adherence to the Nordic Nutrition Recommendations 2023 and all-cause mortality

In this step-by-step tutorial we provide details (including R-code) on each of the six intermediary steps following the specification (step 1) and emulation (step 2) of the target trial protocol, as described briefly in the main paper. Intermediary steps 1.1 to 1.6 represent the specification of all protocol components (from eligibility criteria to statistical analysis) of the hypothetical target trial, and 2.1 to 2.6 represent the emulation process for each of the protocol components. **Table 2** in the main paper contains a summary of the intermediary steps.

---------------------------------------------------------------------------------------------------------------------

**Simulating the synthetic data to be used**

We start by simulating a population of n=10,000 men and women aged 45-60 years of age at the time of recruitment in 2005. Age is not shown in the simulated data. Repeated measures of diet, captured through validated food frequency questionnaires (FFQs), and confounders were assessed every five years (2005, 2010, 2015, 2020, 2025). For simplicity, we simulate complete data on all covariates and no loss to follow-up. We simulate values for a binary outcome (0/1) and three food groups: fruits and vegetables (F&V), red and processed red meat (RPM) and nuts.

The simulated data represent a simplification of the real world and is used for educational purposes only. In practice, additional decisions may be relevant as how to handle missing data, loss to follow-up, competing events, and what confounders to select and adjust for.

#Load packages

library(dplyr)

library(purrr)

library(gfoRmula) #R-package for the parametric g-formula

#Simulating data in long format

#Set seed

set.seed(123)

#Set parameters for the number of IDs and time points

n_ids <- 10000 #n=10000 participants in the cohort

starts <- c(2005, 2010, 2015, 2020) #Start of each follow-up period

stops <- c(2010, 2015, 2020, 2025) #End of each follow-up period

n_intervals <- 4 #Number of intervals

timepoints <- 0:3 #Time points based on the number of intervals

#Baseline variables

baseline <- tibble(

ID = 1:n_ids,

sex = rbinom(n_ids, 1, 0.5), #0=female

education = sample(1:3, n_ids, replace = TRUE, prob = c(0.4, 0.4, 0.2)) #0-9 years=1, 9-12 years=2, >12 years=3

)

#A function for the evolving of time-varying variables

evolve <- function(x0, n, drift = 0, sd = 1) {

x <- numeric(n); x[1] <- x0

if (n > 1) for (t in 2:n) x[t] <- x[t-1] + drift + rnorm(1, 0, sd)

x

}

#Long format data set up

df_long <- baseline %>%

slice(rep(1:n(), each = n_intervals)) %>%

mutate(

timepoint = rep(timepoints, times = n_ids),

start = rep(starts, times = n_ids),

stop = rep(stops, times = n_ids)

) %>%

group_by(ID) %>%

mutate(

#Baseline

#We make sure BMI does not go below 16 and dietary variables no lower than 0

#Dietary variables and BMI are dependent on sex and education

fv0	= pmax(0, rlnorm(1, log(350 + 20*(education[1]-2) - 10*sex[1]), 0.3)),

red0 = pmax(0, rlnorm(1, log(60 - 5*(education[1]-2) + 10*sex[1]), 0.3)),

nuts0 = pmax(0, rlnorm(1, log(20 + 2*(education[1]-2)), 0.4)),

bmi0 = pmax(16, rnorm(1, 25 - 0.6*(education[1]-2) + 0.4*sex[1], 4)),

#Time-varying

#We make sure BMI does not go below 16 and dietary variables no lower than 0

#Dietary variables are dependent on sex, education and BMI but BMI is not dependent on other variables

bmi = pmax(16, evolve(bmi0[1], n_intervals, drift = 0.05, sd = 1)[timepoint+1]),

fruits_veg = pmax(0, evolve(fv0[1], n_intervals, drift = 2*(education[1]-2), sd = 15)[timepoint+1] - 2.0*(bmi - 25)),

red_meat	= pmax(0, evolve(red0[1], n_intervals, drift = 1*sex[1], sd = 8)[timepoint+1] + 1.0*(bmi - 25)),

nuts	= pmax(0, evolve(nuts0[1], n_intervals, drift = 0.2, sd = 3)[timepoint+1] - 0.5*(bmi - 25))

) %>%

ungroup() %>%

select(ID, timepoint, start, stop, sex, education,

bmi, fruits_veg, red_meat, nuts)

#Simulate event

#The probability of having the event is dependent on diet, education, sex and BMI

df_long <- df_long %>%

group_by(ID) %>%

mutate(

lp = -4.2 +

0.03*(bmi - 25) +

0.001*red_meat -

0.0004*fruits_veg -

0.002*nuts +

0.25*sex - 0.12*(education - 1) + 2.5,

p = plogis(lp) %>% pmin(0.6) %>% pmax(0.001),

event = {

died <- FALSE

out <- integer(n())

for (i in seq_len(n())) {

if (!died && rbinom(1, 1, p[i]) == 1) { out[i] <- 1; died <- TRUE } else out[i] <- 0

}

out

}

) %>%

ungroup() %>%

select(-lp, -p)

#Exclude rows after event has developed

df_long <- df_long %>%

group_by(ID) %>%

arrange(timepoint, .by_group = TRUE) %>%

mutate(after_event = dplyr::lag(cummax(event), default = 0)) %>%

filter(after_event == 0) %>%

select(-after_event) %>%

ungroup()

#Print df_long

df_long

#If the data are provided in wide format, the reader is advised to use the *pivot_longer()* function from the ***tidyr*** package to convert the data into long format (e.g., *pivot_longer(data, cols = starts_with("..."), names_to = "...", values_to = "..."))*

---------------------------------------------------------------------------------------------------------------------

**Data structure**

The simulated data is structured in long format, with each time point and ID presented as a separate row (**Supplementary Table 1**). The event (here, all-cause mortality) is coded as zero or one for each row, meaning that when the event occurred for ID 2 between 2005 and 2010, the event variable is coded as one for that row and no further rows are presented. Should the event not occur during a given time interval, the event variable is coded as zero. **Supplementary Table 1** shows the data structure for the two first IDs.

**Supplementary Table 1**. Data structured in long format with ID 1 and 2 as examples.

| ID | timepoint | start | stop | sex | education | bmi | fruits_veg | red_meat | nuts | event |
| --- | --- | --- | --- | --- | --- | --- | --- | --- | --- | --- |
| 1 | 0 | 2005 | 2010 | 0 | 2 | 26.9 | 708.9 | 48.6 | 17.5 | 0 |
| 1 | 1 | 2010 | 2015 | 0 | 2 | 27.2 | 720.0 | 35.7 | 21.8 | 0 |
| 1 | 2 | 2015 | 2020 | 0 | 2 | 28.2 | 711.7 | 38.8 | 19.5 | 0 |
| 1 | 3 | 2020 | 2025 | 0 | 2 | 27.5 | 700.4 | 40.1 | 22.4 | 0 |
| 2 | 0 | 2005 | 2010 | 1 | 2 | 28.2 | 316.8 | 68.8 | 20.5 | 1 |
| … | … | … | … | … | … | … | … | … | … | … |

---------------------------------------------------------------------------------------------------------------------

**The causal question of interest**

Let’s suppose that we were originally interested in investigating whether adhering to the Nordic Nutrition Recommendations outlined in 2023 (NNR23) could lower all-cause mortality risk in Swedish adults. Once the hypothetical target trial had been specified (see points 1.1-2.6 below), the causal question of interest was refined to: *what is the effect of sustained adherence to the Nordic Nutrition Recommendations 2023 (NNR23), as compared to no intervention, on the 20-year risk of all-cause mortality in Swedish middle-aged men and women?*

---------------------------------------------------------------------------------------------------------------------

**Step 1.1 and 2.1 – Defining and emulating the eligibility criteria**

**1.1 (defining the eligibility criteria):** In the trial, men and women aged 45-60 years of age would be eligible to participate.

Ideally, we would include men and women with a wider age distribution, but as the observational data was restricted to those aged 45-60 years, we had to modify the eligibility criteria. This is an example of the iterative process underlying the specification and emulation of a target trial, as illustrated in **Figure 1** in the main paper.

**2.1 (emulating the eligibility criteria):** The eligibility criteria are emulated by restricting the population to men and women aged 45-60 years of age. Participants also need to have valid information on baseline covariates, including diet, BMI, education and sex, to be eligible. As such, the data is restricted to those with complete baseline covariate information, a common approach implemented in target trial emulation studies.

**General points of consideration for 1.1 and 2.1:** Are all individuals between the ages of 45-60 eligible to participate? What about those who cannot adhere to the interventions, e.g., individuals with gastrointestinal issues, vegans, or a nut allergy? In this example, we disregard these, but in practice, these may need to be taken into account.

---------------------------------------------------------------------------------------------------------------------

**Step 1.2 and 2.2 – Defining and emulating the treatment strategies**

**1.2 (defining the treatment strategies):** In the trial, participants would be randomized to either the NNR23 or no intervention. For simplicity, we assume that the NNR23 include only three food groups. Thresholds for the food groups in the NNR23 would be: F&V ≥ 500 grams/day, RPM ≤ 350 grams/week, and nuts ≥ 20 grams/day. At each 6-month follow-up meeting, individuals would be encouraged to continue to eat according to the intervention until the next scheduled visit. The no intervention group would continue as usual.

**2.2 (emulating the treatment strategies):** The same treatment strategies are defined in the emulation. We also need to make the assumption that reported intake from FFQs represents the average intake until answering the next FFQ, loss to follow-up or end of follow-up. The no intervention arm assumes that individuals eat according to their reported intake from the FFQ. If the natural value of intake falls below or above the recommended value, it will be changed to a value compatible with the intervention under each specified strategy. For example, if a participant’s natural intake of nuts is 15 grams per day at time *t*, the value is changed to exactly 20 grams per day at time *t*. However, if a participant’s natural intake of nuts is 22 grams per day at time *t*, the value is maintained at that level. These intervention strategies are called threshold interventions.

An important modification to the hypothetical trial protocol is that participants are followed up every 5 years (due to restrictions on repeated measures in the available data), instead of every 6 months.

**General points of consideration for 1.2 and 2.2:** Is the assumption of sustained adherence between each follow-up reasonable? Are all participants intervened on? What about those who develop gastrointestinal issues or an allergy to nuts? Are there people in the data actually adhering to these intervention thresholds? If not, thresholds probably need to be adapted to the population^1,2^. What is the composition of the diet in the no intervention group? This would be important to describe for correct interpretation and comparison across studies.

#Overall setup of the treatment strategies

#We want to intervene on fruits and vegetables, red and processed red meat (red_meat) and nuts

intvars <- list(

c("fruits_veg", "red_meat", "nuts") #Intervention (NNR23 diet)

)

#We then specify our threshold interventions

#As an example, if a participant consumes 15 grams of nuts, the value is set to 20 grams, but if a participant consumes 22 grams of nuts, the value is not changed

#There are other possibilities to define the intervention strategies in the gfoRmula package

interventions <- list(

list(

c(threshold, 500, Inf), #Fruits and vegetables >= 500 grams/day

c(threshold, -Inf, 50), #Red meat <= 350 grams/week = 50 grams/day

c(threshold, 20, Inf)	#Nuts >= 20 grams/day

)

)

#Description of the diet intervention

int_descript <- c("NNR23 diet")

#Adherence checks at each time point

adherence_by_time <- df_long %>%

mutate(

adhere_fruits_veg = fruits_veg >= 500,

adhere_red_meat	= red_meat <= 50,

adhere_nuts	= nuts >= 20,

adhere_all	= adhere_fruits_veg & adhere_red_meat & adhere_nuts

) %>%

group_by(timepoint) %>%

summarise(

n_adhere_fruits_veg = sum(adhere_fruits_veg),

n_adhere_red_meat	= sum(adhere_red_meat),

n_adhere_nuts	= sum(adhere_nuts),

n_adhere_all	= sum(adhere_all)

)

adherence_by_time #There are people adhering to all components at each time point

#Describe the usual diet over time

#This will give an overall picture of how the usual diet changes over time

no_intervention_arm_descriptive <- df_long %>%

group_by(timepoint) %>%

summarise(

across(

c(fruits_veg, red_meat, nuts),

list(

median = median,

q25 = ~quantile(.x, 0.25),

q75 = ~quantile(.x, 0.75)

)

)

)

no_intervention_arm_descriptive

In the summary dataset *adherence_by_time*, we can confirm that there are people adhering to all food components at each time point over follow-up, thereby reducing the risk of positivity violations. In *no_intervention_arm_descriptive*, we describe the observed data over time, representing the usual diet in the population. Reported mean intakes of the three foods are fairly constant over time. Importantly, the reported intake of nuts is relatively high, which must be considered when interpreting and transporting the results across populations.

---------------------------------------------------------------------------------------------------------------------

**Step 1.3 and 2.3 – Defining and emulating the assignment procedure**

**1.3 (defining the assignment procedure):** In the trial, each participant would be randomly assigned (in a 1:1 ratio) to either the NNR23 or to no intervention in 2005. Participants would be aware of their allocated diet. **2.3 (emulating the assignment procedure):** We assume randomization based on BMI, sex and education at baseline in 2005.

**General points of consideration for 1.3 and 2.3:** Are there any other baseline confounders that need to be adjusted for? What about pre-baseline dietary variables and/or pre-baseline covariates? If all confounders are measured at the same time as the exposure, this needs to be considered.

For this example, we made the strong, and possibly unreasonable, assumption that previous diet was not a relevant confounder. A more valid approach would have been to change the baseline to 2010 to allow for adjustment for pre-baseline diet in 2005. Sensitivity analyses to adjust for BMI from the previous questionnaire could also be performed.

---------------------------------------------------------------------------------------------------------------------

**Step 1.4 and 2.4 – Defining and emulating outcome and follow-up**

**1.4 (defining outcome and follow-up):** In the trial, the outcome would be all-cause mortality. Participants would be followed up from baseline in 2005 to event, loss to follow-up or administrative end of follow-up in 2025.

**2.4 (emulating outcome and follow-up):** The outcome is the same in the emulation. Loss to follow-up is defined as not returning a valid FFQ.

**General points of consideration for 1.4 and 2.4:** Are there any competing events that need to be considered? How are they handled? For all-cause mortality, this is not an issue.

Readers are referred to McGrath, et al.^3^ if competing events are present. The data need to be structured in accordance with the causal estimand of interest. For example, if a particular total effect is targeted in the presence of competing events (the contrast of different counterfactual cause-specific cumulative incidences), the outcome of interest must be coded as “NA” on the same row where the competing event is coded 1. At the same time, a competing event model must be specified.

---------------------------------------------------------------------------------------------------------------------

**Step 1.5 and 2.5 – Defining and emulating the causal contrast**

**1.5 (defining the causal contrast):** In the trial, the per-protocol effect would be the primary causal contrast of interest. The per-protocol effect targets a joint effect of both assignment (through randomization) and adherence to the treatment strategies.

**2.5 (emulating the causal contrast):** The observational analogue of the per-protocol effect is the causal contrast of interest in the emulation.

**General points of consideration for 1.5 and 2.5:** Is there treatment-confounder feedback present for the estimation of the per-protocol effect? If so, g-methods are required. As an example, treatment-confounder feedback occurs when prior diet affects BMI, which in turn influences subsequent dietary adherence. G-methods refer to inverse probability weighting, parametric g-computation, or doubly robust approaches like targeted maximum likelihood estimation.

---------------------------------------------------------------------------------------------------------------------

**Step 1.6 and 2.6 – Defining and emulating the statistical analysis**

**1.6 (defining the statistical analysis):** In the trial, the parametric g-formula would be applied, with adjustment for pre- and post-baseline variables associated with adherence, loss to follow-up and the outcome. In short, the parametric g-formula is a generalized form of standardization used to estimate the risk of the outcome under hypothetical interventions by standardizing over the joint distribution of time-varying covariates^3^.

**2.6 (emulating the statistical analysis):** The same analysis is applied to the emulation. Baseline sex and education as well as BMI as a time-varying covariate will be adjusted for.

**General points of consideration for 1.6 and 2.6:** Are there other important time-varying covariates predictive of adherence and/or loss to follow-up? Are the assumptions of no unmeasured confounding, no measurement error, no selection bias and no model misspecification reasonable? Consider sensitivity analyses to examine robustness to these assumptions.

Sensitivity analyses commonly conducted include the use of negative control outcomes^4^, adjustment for previous dietary variables in a subset of the population^1^, changing the functional forms of the covariates as well as changing the order of the covariates when specifying the covariate models in the parametric g-formula. There are, however, other sensitivity analyses and quantitative bias analyses that could be used to examine robustness to these assumptions ^5,6^.

#Overall set up of the parametric g-formula

id <- "ID"

time_points <- 4 #Number of intervals in data (2005-2010, 2010-2015, 2015-2020, 2020-2025)

time_name <- "timepoint"

nsimul <- 10000 #We simulate a population equal in size to the one observed at baseline

ncores <- as.numeric(parallel::detectCores())

#Specification of the outcome model

#We include sex, education, BMI and lagged values of all time-varying covariates

outcome_type <- "survival"

outcome_name <- "event"

ymodel <- event ~ sex + education + bmi + lag1_bmi +

fruits_veg + lag1_fruits_veg + red_meat + lag1_red_meat + nuts + lag1_nuts + timepoint

#Specification of baseline- and time-varying covariates

basecovs <- c("sex", "education") #Baseline confounders

covnames <- c("bmi", "fruits_veg", "red_meat", "nuts") #Time-varying covariates

covtypes <- c("normal", "normal", "normal", "normal") #Functional forms of time-varying covariates

histories <- c(lagged) #Stores lagged values of the variables in the data set specified for histvars below

histvars <- list(c("bmi", "fruits_veg", "red_meat", "nuts")) #Histories are saved for these covariates

#Specification of covariate models for time-varying covariates

#We assume BMI is not influenced by food, at the same time point, as BMI is a confounder of diet

#We further assume dietary variables are only dependent on previous diet (and confounders)

#We model independent variables as continuous variables but other possibilities exist

covparams <- list(covmodels = c(

bmi ~ sex + education + lag1_bmi + lag1_fruits_veg + lag1_red_meat + lag1_nuts + timepoint,

fruits_veg ~ sex + education + bmi + lag1_bmi + lag1_fruits_veg + lag1_red_meat + lag1_nuts + timepoint,

red_meat ~ sex + education + bmi + lag1_bmi + lag1_fruits_veg + lag1_red_meat + lag1_nuts + timepoint,

nuts ~ sex + education + bmi + lag1_bmi + lag1_fruits_veg + lag1_red_meat + lag1_nuts + timepoint

))

#Parametric g-formula

#Without competing events as the outcome of interest is all-cause mortality

#We set ref_int = 0 as we only have one intervention and compare with no intervention

#We set nsamples = 200 bootstraps but this should preferably be increased for valid estimation of variance

gform_model <- gformula(obs_data = df_long, id = id,

outcome_name = outcome_name, outcome_type = outcome_type, ymodel = ymodel,

time_points = time_points, time_name = time_name,

covnames = covnames, covparams = covparams, covtypes = covtypes, basecovs = basecovs,

intvars = intvars, interventions = interventions, int_descript = int_descript,

histories = histories, histvars = histvars, ref_int = 0,

nsimul = nsimul, parallel = TRUE, nsamples = 200, ncores = ncores, seed = 123)

gform_model

---------------------------------------------------------------------------------------------------------------------

**Extraction of the simulated data set**
To better understand the underlying data-generating process, the simulated data set can be extracted.

#Extract the simulated data set by setting nsamples to 0 and sim_data_b to TRUE

#The simulated data set represents the data-generating process as specified by the parametric g-formula models

gform_model_sim <- gformula(obs_data = df_long, id = id,

outcome_name = outcome_name, outcome_type = outcome_type, ymodel = ymodel,

time_points = time_points, time_name = time_name,

covnames = covnames, covparams = covparams, covtypes = covtypes, basecovs = basecovs,

intvars = intvars, interventions = interventions, int_descript = int_descript,

histories = histories, histvars = histvars, ref_int = 0,

nsimul = nsimul, parallel = TRUE, nsamples = 0, sim_data_b = TRUE, ncores = ncores, seed = 123)

gform_model_sim_data <- as.data.frame(gform_model_sim$sim_data)

gform_model_sim_data

**Supplementary Table 2**. An excerpt of the simulated data showing ID, time point and intake of nuts for each arm.

| natural.course.id | natural.course.timepoint | natural.course.nuts | NNR23.diet.id | NNR23.diet.timepoint | NNR23.diet.nuts |
| --- | --- | --- | --- | --- | --- |
| 1 | 0 | 17.54 | 1 | 0 | 20.00 |
| 1 | 1 | 21.36 | 1 | 1 | 23.82 |
| 1 | 2 | 21.23 | 1 | 2 | 23.69 |
| 1 | 3 | 24.07 | 1 | 3 | 26.53 |

NNR, Nordic Nutrition Recommendations.

From **Supplementary Table 2**, we can see that for ID 1, reported intake of nuts increased over time from 17.54 grams per day in 2005 to 24.07 grams per day in 2020, when hypothetically assigned to no intervention. However, when hypothetically assigned to the NNR23, ID 1 reported consuming 20.00 grams of nuts per day in 2005 and 26.53 grams in 2025. As the threshold intervention for nuts is set to 20 grams/day, ID 1 did not go below this threshold when hypothetically assigned to NNR23. More details, including confounder data, lagged values for each covariate, and predicted risk of the outcome, are shown in *gform_model_sim_data*.

---------------------------------------------------------------------------------------------------------------------

**Results**

The output from the parametric g-formula is shown in **Supplementary Table 3**. The estimated risk difference is -3.3 percentage points (95% CI: -5.2, -1.9) and the estimated risk ratio is 0.93 (95% CI: 0.89, 0.96). The average proportion of individuals who would have been required to change their diet at each time point to adhere to the assigned NNR23 strategy is 77.4%.

**Supplementary Table 3**. Results for the NNR23 and all-cause mortality.

|  | Risk %  (95% CI) | RD %-points  (95% CI) | RR  (95% CI) | Averaged % intervened on |
| --- | --- | --- | --- | --- |
| NNR23 | 47.3 (46.3, 48.3) | -3.3 (-5.2, -1.9) | 0.93 (0.89, 0.96) | 77.43 |
| No intervention | 44.0 (42.0, 45.8) | ref | ref | 0.00 |

CI, Confidence Interval; NNR, Nordic Nutrition Recommendations; RD, Risk Difference; RR, Risk Ratio.

---------------------------------------------------------------------------------------------------------------------

**Interpretation of the results**

For the results presented in **Supplementary Table 3** to be interpreted as the average causal effect of the hypothetical intervention, the assumptions of *conditional exchangeability*, *positivity*, and *consistency* must hold.

1) *Conditional exchangeability*: Conditional on the measured covariates, there are no unmeasured common causes of diet and all-cause mortality.
2) *Positivity*: Within all levels of covariates (and their combinations), there is a non-zero probability of adhering to each intervention strategy at each time point.
3) *Consistency*: Any means at arriving to a particular food group level will lead to the same all-cause mortality risk.

The first identification assumption is often referred to as no unmeasured confounding. We adjusted for sex, education, and BMI, but whether these are sufficient to satisfy the assumption of conditional exchangeability cannot be verified in the data. Instead, subject-matter expertise is necessary to identify relevant confounders to adjust for.

The second identification assumption may be empirically assessed in simpler settings. We confirmed that some participants adhered to the NNR23 intervention strategy at each follow-up time, thereby reducing the risk of positivity violations. However, the more confounders that are included in the analysis, the more difficult it becomes to verify that participants adhere to the intervention strategies within all covariate strata.

The third identification assumption refers to the requirement that the exposures should be sufficiently well-defined. If different versions of the foods have different effects on all-cause mortality, consistency may be violated. For example, within the RPM group, consuming pan-fried processed bacon may be argued to influence mortality risk differently than consuming stewed lean unprocessed red meat. In contrast, eating nuts with the left hand instead of the right would likely not affect mortality risk; an example of treatment variation irrelevance.

For a comprehensive discussion on these assumptions, the reader is referred to “What if” by Miguel A. Hernán and James M. Robins ([Causal Inference: What If (the book) — Miguel Hernán](https://miguelhernan.org/whatifbook)).

In addition to the identification assumptions, no measurement errors and no model misspecifications are assumed. If all these assumptions hold, the results in **Supplementary Table 3** may be interpreted as estimates of causal effects. However, in practice, sensitivity analyses are often required to assess the robustness of the findings to potential violations of these assumptions. Below, we provide a simple approach to evaluating potential model misspecification.

---------------------------------------------------------------------------------------------------------------------

**Evaluation of model specifications**
The parametric g-formula relies on strong assumptions of no model misspecifications. By comparing the non-parametric risk estimate under no intervention with the parametric g-form risk estimate, the potential for model misspecifications may be evaluated. Likewise, by plotting the non-parametric and parametric covariate mean estimates, model misspecifications may be visually examined. In the presence of censoring from loss to follow-up (not an issue in this example), comparing natural-course risks estimated via inverse-probability weighting with those from the parametric g-formula may be performed^7^.

#Evaluation of model specifications

#From gform_model, the non-parametric (NP) risk estimate under no intervention (0.4725) is similar to the parametric g-form risk estimate (0.4727)

#We can also plot the non-parametric and parametric covariate estimates

plot(gform_model)

The non-parametric risk estimate under no intervention (0.4725) is similar to the g-form risk estimate (0.4727), which supports, but does not guarantee, the absence of gross model misspecification. Plotting the two mean estimates for bmi, fruits_veg and nuts showed moderate agreement, although the mean estimate for red_meat diverged at time point 2 and forward (**Supplementary Figure 1**). Other sensitivity analyses to evaluate model misspecifications would be to change the order of covariates in the model or by changing the functional forms of included covariates.

**Supplementary Figure 1**. Parametric g-formula vs non-parametric natural course covariate mean estimates for the main analysis.

---------------------------------------------------------------------------------------------------------------------

**References**

1.	Ibsen, D. B., Chiu, Y.-H., Gémes, K. & Wolk, A. Hypothetical 22-Year Intervention With DASH Diet Lowered Risk of Heart Failure in a General Population. *Am. J. Epidemiol.* (2023) doi:10.1093/aje/kwad181.

2.	Stern, D. *et al.* Evaluating the 2023 Mexican Dietary Guidelines for Type 2 Diabetes Prevention: A Target Trial Emulation in Mexican Women. *Am. J. Epidemiol.* (2026) doi:10.1093/aje/kwag037.

3.	McGrath, S. *et al.* gfoRmula: An R Package for Estimating the Effects of Sustained Treatment Strategies via the Parametric g-formula. *Patterns* **1**, 100008 (2020).

4.	Lipsitch, M., Tchetgen Tchetgen, E. & Cohen, T. Negative Controls. *Epidemiology* **21**, 383–388 (2010).

5.	McGee, E. E. *et al.* Estimating the Effects of Lifestyle Interventions on Mortality Among Cancer Survivors: A Methodologic Framework. *Epidemiology* **36**, 705–718 (2025).

6.	D’Agostino McGowan, L. Sensitivity Analyses for Unmeasured Confounders. *Curr. Epidemiol. Reports* **9**, 361–375 (2022).

7.	Chiu, Y.-H. *et al.* Evaluating Model Specification When Using the Parametric G-Formula in the Presence of Censoring. *Am. J. Epidemiol.* **192**, 1887–1895 (2023).
